# Physicians’ perspectives on Extreme Risk Protection Orders (ERPOs) in the clinical setting: Challenges and opportunities for gun violence prevention

**DOI:** 10.1371/journal.pone.0274489

**Published:** 2022-09-13

**Authors:** Ashley Hollo, Amy VanderStoep, Shannon Frattaroli

**Affiliations:** 1 Johns Hopkins Bloomberg School of Public Health, Baltimore, Maryland, United States of America; 2 Johns Hopkins Center for Gun Violence Prevention and Policy, Johns Hopkins Bloomberg School of Public Health, Baltimore, Maryland, United States of America; Uniformed Services University of the Health Sciences, UNITED STATES

## Abstract

**Background:**

Firearm-related injuries remain a heavy public health and clinical burden in the United States. Extreme Risk Protection Order (ERPO) laws, which create a path through a civil court process to temporarily remove firearms from individuals deemed to be at risk of harming themselves or others and are one strategy designed to reduce firearm violence. Maryland was the first state to authorize clinicians as ERPO petitioners.

**Objective:**

We aim to document a sample of Maryland physicians’ perspectives about the utility of, any barriers to, and other thoughts on clinicians as ERPO petitioners.

**Design:**

A series of semi-structured interviews with Maryland physicians identified through a combination of purposive and snowball sampling. We coded the transcribed interviews and analyzed the coded transcripts for themes using deductive content analysis.

**Setting/Participants:**

13 Maryland-based physicians interviewed over Zoom in and around Baltimore City, Maryland.

**Results:**

The interviewees had overall positive feedback about ERPO as a gun violence prevention tool in the clinical setting. They identified several barriers to effective implementation such as time spent on paperwork and in court, a lack of awareness among clinicians about ERPO, threats to therapeutic alliance, and a sense of futility in a culture where firearms are easy to obtain. Solutions such as providing clinician education about ERPO laws, allowing for virtual court testimony, and creating a consult service with ERPO specialists to manage ERPO petitions were discussed.

**Limitations:**

This study includes a small sample of Maryland-based physicians.

**Conclusions:**

The physicians we interviewed expressed interest in knowing more about ERPO laws and emphasized education as an important tool for improving implementation. Addressing physicians’ concerns about ERPO implementation will improve their ability to be effective and efficient petitioners.

## Background

Firearm-related injuries are a significant source of morbidity and mortality in the United States. In 2020, firearm-related suicides (n = 24,292) and homicides (n = 19,384) contributed significantly to injury-related deaths. [1, 2] and the total of 45,222 firearm-related deaths in 2020 represents a sharp rise, particularly in firearm homicide [1, 3, 4]. Since the start of the pandemic, the number of firearms entering circulation has increased. Of the days and weeks with the highest number of federal firearm background checks, a proxy for firearm purchases, more than half occurred in 2021 [5]. Among these increases in firearm sales, first-time gun owners represent an estimated 23% of purchasers in 2020 [6]. As the rate of guns in circulation and gun owners increases, strategies for removing guns from people who become prohibited or are at risk of violence are needed.

Policies that temporarily prohibit gun purchase and possession by respondents to domestic violence protection orders (DVPO) are associated with reductions in intimate partner homicide [7]. Extreme Risk Protection Order (ERPO) laws expand DVPO policy by establishing a civil process for law enforcement and other named parties to petition the court for an order that temporarily prohibits firearm possession and purchase for individuals who are behaving dangerously and at risk of suicide or interpersonal violence [8]. ERPO is law in 19 states and the District of Columbia [9]. Early studies suggest that ERPO laws offer a promising strategy for preventing suicide [10].

There are certainly resources available to clinicians to aid in addressing firearms with their patients, including communication strategies [11], access to lethal means training [12], safe storage counseling [13], as well as more comprehensive materials for clinicians [14]. While attention to physician counseling about firearms and strategies for safe storage and voluntary dispossession are increasingly common (and likely a more common clinical response when a patient is at risk of committing violence), there is limited guidance about clinicians’ roles in ERPO implementation. Recent recommendations for clinical education priorities include identifying at-risk individuals and increasing awareness of state policies and programs such as ERPO laws [15]. Maryland was the first state to include clinicians as ERPO petitioners in 2018 [16]; Connecticut, Hawaii, New York, and the District of Columbia also include clinicians as authorized petitioners, although the types of clinicians named in each of these laws varies [9].

A Maryland survey of emergency medicine, pediatrics, and psychiatry physicians at one hospital highlights several barriers to a clinician’s ability to petition for an ERPO, including lack of familiarity with the law, time constraints, and concern about relationships with patients. Of the surveyed clinicians, 71.7% were “not at all familiar” with ERPOs, 62.6% and 70.3% identified time to complete paperwork and time to attend court hearings, respectively, as barriers to petitioning; and 39.6% felt “it may negatively affect [their] relationship with the patient” [17]. In addition to knowledge gaps and administrative burdens, others have noted that few liability protections are in place for clinicians who file ERPOs—or those who fail to file [18]. These barriers need to be addressed if clinicians are to serve as ERPO petitioners. Past survey participants most commonly selected working with a clinical coordinator to manage the ERPO process, training, participating in court hearings remotely, and having access to legal counsel as strategies to address the identified challenges to using ERPO in the clinical setting [17]. We are aware of one online training that is available for clinicians about ERPO, and guidance for legislators about clinicians as ERPO petitioners, however additional efforts are needed to support this aspect of ERPO practice [19, 20].

Expanding on the work of the past clinician survey [17], we sought to gain a deeper understanding of clinician perspectives in order to inform implementation of ERPO in the clinical context in Maryland and similar jurisdictions where clinicians are authorized ERPO petitioners. We developed an interview guide to aid in understanding ERPO as a tool in the clinical setting and subsequently interviewed physicians about ERPOs and their use as a gun violence prevention tool.

## Methods

To inform ERPO implementation in the clinical setting, we conducted a series of key informant interviews with Maryland physicians working in and around Baltimore City. We sought interviewees from the departments of emergency medicine, pediatrics, and psychiatry as these departments are likely to use ERPO and are the specialties included in the earlier Maryland survey.

### Participants

We sampled participants using a combination of purposive and snowball sampling methods, beginning with one clinician from each specialty who has a history of involvement in ERPO research (purposive) and asking them to refer us to additional people to interview (snowball).

### Data collection

We developed an interview guide around four domains informed by the literature and the study goals: 1. Knowledge of and experience with ERPO laws and petitions, 2. Clinician perspectives on ERPO use in their clinical setting(s), 3. Potential barriers to clinicians as ERPO petitioners, and 4. Whether a designated ERPO clinical coordinator could address the previously discussed barriers. One author (AV) conducted the interviews via Zoom using the guide and recorded the interviews with participants’ permission. Each of the four domains had several possible probing or follow-up questions. Based on where the respondent led the interview, not every question was asked of every respondent.

### Data analysis

We used the Zoom Cloud Recording transcripts feature to obtain transcripts of the interviews. Our methodology was most consistent with deductive thematic analysis, although we were open to inductive findings. Two authors (AM and AV) reviewed the transcripts to correct any voice-to-text transcript errors, and to obtain a general sense of the data provided through the interviews. We then created a codebook from the interview guide based on the domains and questions (deductive) and added codes based on interviewees’ responses (inductive). The final codebook included definitions of each code, guidance as to when to apply and not apply each code, and examples of coded text for each code. We discussed, reviewed and revised the codebook as we gained familiarity with the data. Two authors (AM and AV) double-coded two transcripts in Microsoft Word, compared how they applied the codes, and discussed any discrepancies to increase the consistency of coding between them. We discussed the coding process during our regular team meetings in order to practice iterative analysis and engage in reflection in keeping with best practices for qualitative research methods [21]. Throughout the iterative analysis, we evaluated whether new ideas were emerging and stopped data collection once we agreed we reached saturation.

### Reflexivity statement

The authors undertook this research with different experiences that informed their roles and understanding of the subject under study. The co-first authors (AH, AV) were enrolled in a joint MD/MPH program at the time they collected these data. Injury prevention and gun violence prevention were focus areas of their MPH studies and both sought out opportunities to learn more about clinician-initiated ERPO use. The third author (SF) has previously published on ERPO policy generally and ERPO use in clinical settings specifically and was the lead author on the publication detailing the survey findings on which the current study is based. While the first co-authors were students at the institution where some of the interviewees worked, they had no relationship with them prior to conducting the interviews for this study. The third author collaborates with three of the interviewees and introduced the two first authors to them for the purposes of this study. The third author did not participate in any of the interviews.

### Ethical considerations

This study was deemed to be non-human subjects research by the Johns Hopkins Bloomberg School of Public Health Institutional Review Board on November 4, 2020. Participants were told at the beginning of the interview Zoom call that the purpose of the interview was to study clinicians’ perspectives on extreme risk protection orders and the goal was publication of the results. Prior to starting the zoom recording, they were verbally asked if they consented to their responses being recorded. All participants consented and if any had said no, the interview would have ended at that point.

## Results

We conducted 13 semi-structured interviews with 6 psychiatrists, 3 emergency medicine physicians, 2 pediatric emergency medicine physicians, 1 pediatrician, and 1 pediatric psychiatrist between April and May 2021. Of the 11 who provided demographic data, 7 identified as women and 4 as men with mean age of 44 (34–57). They had an average of 12.5 years in practice (5–27) with an average of 10.6 of those years (5–27) practiced in Maryland. All were licensed to practice medicine in Maryland, and all but one were actively practicing when interviewed. Both the interviews and results were organized based on the pre-determined four domains described in the methods: 1. Knowledge of and experience with ERPO laws and petitions, 2. Clinician perspectives on ERPO use in their clinical setting(s), 3. Potential barriers to clinicians as ERPO petitioners, and 4. Whether a designated ERPO clinical coordinator could address the previously discussed barriers.

### Knowledge of and experience with ERPO laws and petitions

The breadth and depth of ERPO knowledge among interviewees varied, ranging from those who were unaware of the law (2/13) to those who are currently engaged in ERPO research and/or advocacy (3/13). One noted they had heard of ERPO but were unsure of what they were, and the remaining participants (7/13) had heard of ERPO and were familiar with the basics of the legislation. All interviewees agreed that their clinical colleagues did not have the knowledge needed to use ERPO in their clinical practice. None of the respondents had petitioned for an ERPO at the time of their interview, although a few had heard of colleagues petitioning and some had spoken to these colleagues about their experiences.

### Clinician perspectives on ERPO use in their clinical setting(s)

Interviewees agreed and cited reasons that ERPOs should be a tool at their disposal. Some mentioned that patients often express concerns to their clinicians that they may not share with family members, reinforcing a general idea expressed that physicians, unlike law enforcement, may have close relationships with their patients that facilitate trust. This trusting relationship aligns with ERPO as a civil (not criminal) process designed to protect and not punish respondents. As one interviewee explained, “*I think it’s more helpful to have this very clinical*, *life-saving measure being sponsored by or initiated by a clinician*, *who is generally on your side*. *It’s best for these things to be seen as a life-saving measure and not a punitive or potentially stigmatizing thing*, *as might be the case when something is initiated and carried out by the police*, *especially in communities of color*.*”*

The potential for ERPO as a tool for intervening prior to mass shooting and suicide threats, was evidence enough for one clinician to support ERPO use by clinicians. An emergency medicine physician expressed their support for ERPO given that the care they provide can involve patients in duress who are contemplating violence. Assessing risk is part of the job that clinicians are trained to do, and several interviewees connected their risk assessment skills to the risk of violence that is necessary to establish in an ERPO petition. Additionally, clinicians described their moral and ethical responsibility to protect their patients from harm and presented filing for an ERPO as an extension of that responsibility. Some interviewees drew parallels between ERPO and emergency petitions (EPs), noting that both involve temporary suspension of rights, but that EP petitioners do not need to appear in court, making the process more feasible in an emergent situation and more compatible with busy clinical schedules.

Across specialties, interviewees shared that another specialty might be better equipped to be the ERPO petitioner. For example, emergency medicine physicians reasoned that it is more fitting for psychiatrists to petition for an ERPO because a patient in crisis will often have their care transitioned to a psychiatrist who will oversee their discharge planning. Similarly, outpatient psychiatrists described inpatient or emergency medicine physicians as the ideal petitioner since they assess patients while they are in crisis. Finally, interviewees working mostly in emergency and inpatient settings noted that outpatient clinicians have better rapport with their patients and could be more effective petitioners.

Finally, despite overall positive attitudes towards clinician petitioning, several interviewees noted that an ERPO may not be the best immediate solution for patients in a crisis, given the court processes and amount of time involved. A faster response, such as inpatient hospitalization, may provide a more direct, definitive, and effective intervention. Multiple interviewees commented that ERPO petitions may hold the greatest clinical potential in inpatient psychiatric discharge as restricting access to lethal means may increase patient and provider comfort with discharge, and may even allow for earlier discharge, with one clinician citing evidence that the month following inpatient psychiatric discharge is one of the highest risk periods for suicide.

### Potential barriers to clinicians as ERPO petitioners

Throughout the interviews, six main barriers were identified. These are described here as well as in Table 1, with associated example quotations.

**Table 1:** Barriers to clinicians petitioning for ERPO.

**Lack of knowledge or awareness**	“Some clinicians don’t understand the law and it’s certainly not something that we’re educated about in training” “I think that most clinicians don’t even know that they could petition. They don’t know scenarios in which they should be considering [ERPOs], in which they might be applicable. They don’t know that process to make a petition”
**Therapeutic alliance**	“Other barriers…the worry about therapeutic alliance, this very legitimate worry, especially for outpatient [clinicians], you actually have a relationship with them. If they know that you can take away their gun and they don’t want that to happen, they might be less forthcoming” “The first principle by which we’re bound is do no harm, and I would argue that restriction of access to legal means when somebody is truly at risk of harm is sort of the ultimate expression of do no harm. So yes, you have to break a few eggs. You might impact your rapport with the patient. But in a cost-benefit analysis, if you truly believe that’s the right thing to do, you need to do it”
**Time commitment**	“…you’d be surprised at how unsupportive of [court appearances] a hospital administrator would be…but yeah that’s probably the most common barrier to somebody carrying [an ERPO] out is you simply can’t get to court.” “But I think the barrier’s just time of actually having to complete those in-person court appearances as a petitioner”
**Urgency of situation**	“I worry a little bit about something that involves two court visits not happening particularly quickly so I’m not sure in the immediacy of the moment it’s going to help. We tend to see patients when they’re in a crisis and we need a solution now not the solution in a few weeks.”
**Gun culture and gun accessibility**	“…I think that access to firearms in Baltimore city is like access to cupcakes in New York…. You don’t have to walk two blocks…I think taking someone’s gun away in Baltimore City doesn’t necessarily have the same protection that it might for somebody who doesn’t have such immediate access” “Especially with firearms, there’s a huge cultural component to familiarity with and comfort with and ownership of firearms…I think there are a lot of areas where, culturally firearms are hugely accepted part of everyday life and I think for clinicians in those areas, it may be really difficult to overcome the cultural context of that and to imagine utilizing an ERPO”
**Fear for safety or repercussions**	“I think on an individual level, it can feel risky.. you know patients if [the patient] or their families hold tightly to these second amendment concepts, they know where you work and it’s not hard with the internet to figure out where people live. And there is a certain amount of fear in aggravating…in not knowing how zealously people hold on to their gun right.” “If you don’t file the petition, can you be sued…that’s one of the concerns, I think. I don’t think a psychiatrist or any clinician should be punished if they didn’t file the petition”

Many expressed their sense that even among colleagues who are aware of ERPO law, few know enough to be comfortable petitioning and described familiarity with nuances about the law that affect uptake. One specific knowledge gap discussed was the issue of firearm access versus firearm ownership among pediatric patients. Some of the clinicians who treat pediatric patients expressed confusion about whether these laws apply to them as many of their patients have access to firearms but are not the legal owners of the firearms, as they are not yet of legal age. This lack of clarity about whether the ERPO possession prohibition for a minor respondent would apply to their parent or guardian’s firearms is an area where some interviewees expressed a need for guidance.

Several interviewees stated that filing an ERPO petition may damage the therapeutic alliance they have built with their patients. This was not as large of a concern among emergency medicine physicians who do not have longitudinal patient relationships.

Interviewees also mentioned time as a significant barrier. In addition to completing the initial petition, petitioners must be present in the courtroom for two hearings. Several noted that the amount of paperwork and extra responsibility is already increasing in the clinical setting, and this would be one more thing on their already full plate. Interviewees also expressed these concerns about time and being shorthanded and overworked during the COVID pandemic.

Interviewees brought up concerns about two types of bias that could impact the petitioning process. Individual clinician bias could impact who is a respondent (are clinicians more likely to consider ERPO for some patients than others), while bias among law enforcement could impact the firearm removal process. Law enforcement is responsible for serving ERPOs issued by the court and should dispossess respondents of firearms as part of that service process. Some interviewees voiced concerns about that dispossession interaction. In the words of one interviewee “*there’s always the risk that you can have somebody who we perceive to be a threat to themselves*, *and you send the police in to remove their firearms and it results in an escalation where something bad happens*.”

Some commented on the futility of removing firearms from individuals who live in areas where they have ready access to other firearms. Urban areas were specifically mentioned in this context, “*They don’t have access to guns*, *but they’ll say they can go get one off the street and harm themself at some point*.” Recognition that ERPO is one strategy for reducing access to lethal means that does not address the larger context in which respondents live was prominent in the data.

Gun ownership is a highly politicized topic, and some interviewees expressed concern about retaliation or other repercussions to their safety associated with petitioning, or not petitioning. They described not wanting to put themselves or family members at risk by conflict with individuals with whom they filed an ERPO petition against. Interviewees also described a potential unintended consequence of patients withholding information out of concern that their clinician would use that information as part of an ERPO petition.

Additional, relevant thoughts on petitioning barriers from interviewees available in Table 1.

### Addressing the barriers and evaluating the role of a clinical ERPO coordinator

Interviewees described the structured education time built into the profession as an opportunity to ensure clinicians are current about ERPO law. Using Continuing Medical Education (CME) credits, holding in-service talks on ERPO, and updating clinical staff on any uses or changes to the law are all strategies offered by interviewees in response to the need for a more informed clinical community. In addition, creating a website and helpline for petitioners would provide an accessible resource that is immediately responsive to questions and provides support that interviewees anticipate clinician petitioners will need.

Interviewees also raised how legislative changes could increase physician uptake of ERPO by simplifying the process for clinicians. Changing the law to allow for virtual court appearances so clinicians do not have to leave their place of work to go to court was cited as a time saver that is consistent with evolving court practices under COVID. Interviewees also looked to the EP and psychiatric certification processes as models for clinician use of ERPO, describing the convenience of having administrative judges on site to hear these requests, as happens in Maryland. Lastly, automatic ERPOs were suggested for people in certain scenarios, for example any person admitted for a suicide attempt could qualify for an automatic ERPO, regardless of current firearm possession, thereby reducing the decision and time burdens for the clinical team.

There were two main hospital-level recommendations to address clinician time constraints. The first was to include ERPO in the hospital/clinic protocols to automate and streamline petitioning. The second was to have a dedicated specialist or consult team to facilitate the ERPO process so the treating physician can focus on the immediate care needs and their other clinical responsibilities. Interviewees pointed to child abuse response coordination teams that exist in some institutions, where a dedicated team is called in to evaluate and document suspected child abuse while also managing communication with the legal system. Interviewees described this type of consultation as one that could be organized through the electronic medical record system with a team that was on-site or virtual.

The response from clinicians regarding ERPO specialists was overall positive. Some interviewees shared concerns about dedicated ERPO specialist not having the appropriate clinical knowledge or risk assessment insight about individual patients to accurately and adequately convey the behaviors that prompted the EPRO petition to presiding judges. Interviewees described these concerns as surmountable by including ERPO specialist petitioners into daily patient rounds and involving them throughout the patient encounter. As interviewees reflected on the different clinical locations in which ERPO petitions would be useful (large academic hospitals versus small outpatient clinics) they noted it is not feasible to have an in-person specialist at every clinical site. For this reason, it was suggested that ERPO specialist support could be run by a larger health system or third party (i.e. health department). This type of consult service could travel to multiple sites or be reached over the phone and would provide clinicians with more equitable access. One interviewee stated, “*you can create regional or citywide teams…that you can consult with and have somebody on call for the city and they can consult and make those kinds of assessments in real time within healthcare settings*.*”*

After implementing a consult/specialist service, interviewees still noted that such intervention does not necessarily fix the lack of knowledge/awareness among clinicians, and that education is central to effective use of ERPOs in the clinical setting.

## Discussion

This is the first qualitative study documenting clinician perspectives about clinicians as ERPO petitioners, and it offers insight into the barriers to this intervention and potential strategies to address those barriers from the perspective of physicians. We acknowledge that clinicians often have discussions with patients pertaining to firearm safety and ownership and that there are other interventions clinicians can use to engage with patients prior to attempting firearm removal. The focus of this study and subsequent discussion is the novel approach of implementing ERPOs.

### Education and ERPOs

Education was discussed as a solution to the lack of knowledge about ERPO among clinicians. CME, in-service training, and grand rounds, as mentioned by interviewees, could address the need for education. Furthermore, education could be wrapped into the role of an ERPO clinical specialist. A study published in 2021, highlighting topics for educating clinicians on firearms, is a useful tool, as is the online ERPO training available for CME credit [15, 19]. Such educational efforts should provide training about ERPOs overall and address some of the nuances interviewees raised that are likely to be of particular interest to clinicians. The question of minors and how ERPO applies to situations in which minor patients are not gun owners but have access to guns is one example. Guidance for clinicians and legislators is available [8, 20], but more widespread dissemination of these resources is needed.

Similarly, interviewees suggested that ERPO petitioning may negatively impact the therapeutic alliance. This is a discussion point that can also be a part of ERPO educational efforts and can integrate medical ethics and patient confidentiality into the resulting products. Drawing from established principles and experiences with emergency petitioning can provide useful parallel examples.

### Time and ERPO clinical specialists

As was found in the Maryland physician survey [17], time was frequently raised as a barrier to clinician petitioning. Regardless of how streamlined the ERPO petitioning process becomes, it will always require some time commitment from clinicians. The COVID-19 pandemic has allowed virtual court appearances to become more commonplace. Continuing these practices could provide a mechanism for more efficient clinician petitioning. A more comprehensive solution discussed by interviewees was incorporating ERPO specialists into clinical teams. This would alleviate the time burden, create specialists who can promote awareness and education of ERPO laws, and establish consistent processes for using ERPOs throughout the health care system so that petitions would be less dependent on the primary health care provider.

All interviewees described ERPO petitioning as an important tool for the health care setting, but interviewees were divided on which specialty is best suited to petition. Inpatient psychiatry was most commonly noted as the ideal specialty to handle ERPO petitions as they are often responsible for discharging patients who present at high risk of violence to themselves or others. Understanding which clinical specialties are going to lead ERPO petitioning will likely help promote uptake of ERPO in the clinical setting. Of course, the ERPO clinical specialist model allows for a more diffuse organizational home for ERPO efforts if these new positions are available across clinical departments.

### Legislative considerations

Virtual testimony as a substitute for in-person court appearances offers one way to streamline the ERPO petition process while maintaining due process protections. Interviewees discussed providing clinicians with the option of testifying virtually for ERPO hearings as an easy solution for making ERPO petitioning more feasible for clinicians. Another interviewee questioned whether a court hearing is always needed to decide an ERPO petition. For patients admitted following a suicide attempt, they have established their risk for violence through their violent actions perhaps rendering a court hearing superfluous. These and other changes for the ERPO process to be better aligned with clinical demands would likely require changes to ERPO policy.

### Limitations

This study provides an initial examination of physician perspectives about ERPO in one region of one state. As such, it provides insights into how in states where clinicians are authorized ERPO petitioners, implementation of ERPO can realistically occur, but does not offer generalizable findings. We acknowledge that the scope of the study was defined by the specific nature of both the interviewed population (Maryland clinicians who were interested in discussing firearm policy) and the interview guide, which was informed by past survey data [17]. Such sampling decisions reflect the purposive nature of qualitative research and the acknowledged value of seeking out information rich interviewees who can best inform the research topic. This scope and respondent population does not result in generalizability. The study was designed to inform the theoretical understanding of ERPO use in the clinical setting and as the first qualitative exploration of this topic it accomplishes that goal. Importantly, there remains much work to be done in order to understand ERPO implementation in the clincial setting. This study provides a foundation to inform future studies, including different designs to achieve generalizability.

## Conclusion

Based on this small sample, there is evidence to suggest that physicians in multiple medical specialties are open to the use of ERPO in the clinical setting, however, there are barriers to realizing uptake as articulated by the interviewees. Their insights about these barriers offer nuanced insights that can inform implementation efforts and provide concrete suggestions for how to address the identified barriers. As such, these perspectives can inform future programming aimed at increasing knowledge and use of ERPOs in the clinical setting and ensuring that such efforts are mindful of implementation barriers and account for ERPO in the context of the full range of physician options for intervening when patients are at risk of committing violence, including counseling, safe storage and voluntary dispossession. Future studies should focus on expanding the pool of physicians included to more medical specialties within various regions (rural, suburban, urban), clinicians other than physicians who are authorized to petition for ERPOs, and who practice in different clinical settings (e.g. hospitals, community centers, individual office practices). As clinicians grow more experienced with ERPO petitioning, future research should also include individuals who have petitioned for ERPOs in order to add their experiences to the literature that will continue to inform gun violence prevention policy and practice.

## Supporting information

S1 File (PDF)
