# Supplementary material for: Physicians’ perspectives on Extreme Risk Protection Orders (ERPOs) in the clinical setting: Challenges and opportunities for gun violence prevention
Source: PLoS One. 2022 Sep 13;17(9):e0274489. doi: 10.1371/journal.pone.0274489 (PMC9469965; doi:10.1371/journal.pone.0274489)
Supplement: S1 File — (PDF) [file pone.0274489.s001.pdf]

Information collected about each respondent:
Specialty: *[Clinicians within Pediatrics, Emergency Medicine, Pediatric Emergency Medicine, Psychiatry, Pediatric Psychiatry]*
Practice setting(s): *[Outpatient vs. Inpatient practice]*

Interview Questions / Interviewer Guide
Introduction: (Summary from box above) Why this study. What happens to results. How it will be reported. Recorded responses but no names reported. Questions prior to beginning?

1. **Topic:** Gaining a sense of understanding about the clinicians' knowledge of and experience with ERPO laws:
    a. Initial Prompt: Today I would like for us to talk about a relatively new approach to curbing firearm-related injury. Could you start by telling me what you know about Extreme Risk Protection Order or ERPO laws? *(if no background knowledge, move on to #2)*
        i. How did you learn about ERPO laws? (news vs clinical conversations, etc.)
    b. (*If some background knowledge in b*) What do you know about ERPO laws in the state of Maryland?
    c. What is your sense of other clinicians in your specialty or department knowledge about or awareness of ERPO?
    d. Have you or anyone you work with been an ERPO petitioner as a clinician?
        i. If yes, could you talk about that experience?
2. **Topic:** Determining perspectives on the utility of ERPO in the clinical setting:
    a. Background (*read, regardless of participant's prior knowledge*): Extreme Risk Protection Order or ERPO laws allow specific groups of people (law enforcement in all states, family and partners in most states) to petition the court for an order that would temporarily prohibit firearm purchase and possession by someone who is behaving dangerously and at risk of committing violence. The violence includes harm to self, as well as interpersonal violence. ERPO laws now exist in 19 states and the District of Columbia. In Maryland, Hawaii, and the District of Columbia, physicians and other authorized clinicians are able to be ERPO petitioners for patients who they believe pose a risk of harm to themselves or others.
    b. Now that you know a little more about ERPOs [OR: Considering your background knowledge], what are your thoughts on this approach to gun violence prevention?
        i. *If thoughts are mostly of concern*: Can you think of any benefits that may be associated with clinical ERPO petitions?
        ii. *If thoughts are mostly interested or positive*: Can you think of any general situations where a patient expresses concern of harm to self or others and an ERPO would be ill advised or harmful?
        iii. How do you feel about the role of clinicians in this ERPO process? Do you feel the clinical setting is an appropriate setting for this process to be initiated? (*If feels this is not appropriate, probe into clinician v. clinical*

setting as the problem. Why is this not an appropriate actor/setting for ERPOs?)

iv. (*If no comment on ERPO in clinical setting, probe further into this*) In Maryland, where clinicians are able to function as a petitioner, what are your thoughts on the use of this tool? Is this something you foresee as useful in your hospital/practice?

c. Reflecting on your experience and looking to the future, are there situations you can foresee in which you would be inclined to utilize your role as an ERPO petitioner?

i. *Probing:* In general, thinking about your work days/shifts, how frequently do you encounter patients at risk of violence or suicide for whom you may consider an ERPO? (not specific patients)

ii. [If clinician brings up situation where patient is not the one an ERPO would be filed against (other person at risk of potentially harming patient or harming themselves – would need to be handled through family or law enforcement), capture frequency of this situation vs the patient being the potential ERPO respondent, but no need to probe further]

d. Transition: If a patient presents at risk of harming themselves or others, clinicians in Maryland have three options: talk to the patient's family, engage with law enforcement, or initiate the petition themselves. For the rest of our time together, we're going to focus on this third option of clinicians as the petitioner.

3. **Topic:** Evaluating potential barriers to clinicians as ERPO petitioners:

a. What, from your perspective, are the barriers to clinicians using ERPO when presented with a patient who is behaving dangerously and at risk of harm?

i. *If uncertain of barriers or having difficulty listing possible barriers, probe by asking if any of the following would prevent them from functioning as an ERPO petitioner:* knowledge about ERPOs, time to complete paperwork, time to attend courthouse hearing(s), not billable, relationship with patient (therapeutic alliance)

4. **Topic:** Gaining insight into how clinicians see an ERPO navigator functioning in their clinical workplace(s):

a. Focusing on the barriers we discussed, do you have any thoughts on how best to address these barriers?

b. Background: One solution brought forth is to designate someone on staff to serve as an "ERPO specialist" or "ERPO navigator". This individual would be brought into situations where an ERPO petition is being considered to take the lead on the paperwork and be the court hearing representative.

i. What are your thoughts on this idea?

c. Do you have any ideas on how to make this viable, from the perspective of your clinical setting(s)? What would you want this position to look like?

i. *Probe:* How could an ERPO navigator best integrate into the clinical flow?

d. If an ERPO navigator was in place at your clinical workplace, do you foresee any additional barriers to your ability to be an ERPO petitioner?

5. Is there anything else on the topic of ERPO laws or petitions that you would like to discuss or other concerns you have?
6. Is there anyone else I should be talking with who would have good insight into these questions?
